# Mitochondrial Hyperactivation and Enhanced ROS Production are Involved in Toxicity Induced by Oncogenic Kinases Over-Signaling

**DOI:** 10.3390/cancers10120509

**Published:** 2018-12-12

**Authors:** Monica Ceccon, Mario Mauri, Luca Massimino, Giovanni Giudici, Rocco Piazza, Carlo Gambacorti-Passerini, Luca Mologni

**Affiliations:** 1 Department of Medicine and Surgery, University of Milano-Bicocca, 20900 Monza, Italy; mario.mauri@unimib.it (M.M.); rocco.piazza@unimib.it (R.P.); carlo.gambacorti@unimib.it (C.G.-P.); luca.mologni@unimib.it (L.M.); 2 Division of Neuroscience, San Raffaele Scientific Institute, 20132 Milano, Italy; luca.massimino@unimib.it; 3 Tettamanti Research Centre, Pediatric Clinic, c/o Nuovo Osp.San Gerardo, 20900 Monza, Italy; g.giudici@asst-monza.it

**Keywords:** cancer, oncogene, inhibitors, personalized medicine, targeted therapy

## Abstract

Targeted therapy is an effective, rational, and safe approach to solid and hematological tumors treatment. Unfortunately, a significant fraction of patients treated with tyrosine kinase inhibitors (TKI) relapses mainly because of gene amplification, mutations, or other bypass mechanisms. Recently a growing number of papers showed how, in some cases, resistance due to oncogene overexpression may be associated with drug addiction: cells able to proliferate in the presence of high TKI doses become also TKI dependent, undergoing cellular stress, and apoptosis/death upon drug withdrawal. Notably, if a sub-cellular population survives TKI discontinuation it is also partially re-sensitized to the same drug. Thus, it is possible that a subset of patients relapsing upon TKI treatment may benefit from a discontinuous therapeutic schedule. We focused on two different hematologic malignancies, chronic myeloid leukemia (CML) and anaplastic large cell lymphoma (ALCL), both successfully treatable with TKIs. The two models utilized (LAMA and SUP-M2) differed in having oncogene overexpression as the sole cause of drug resistance (CML), or additionally carrying kinase domain mutations (ALCL). In both cases drug withdrawal caused a sudden overload of oncogenic signal, enhanced mitochondria activity, induced the release of a high amount of reactive oxygen species (ROS), and caused genotoxic stress and massive cell death. In LAMA cells (CML) we could rescue the cells from death by partially blocking downstream oncogenic signaling or lowering ROS detrimental effect by adding reduced glutathione.

## 1. Introduction

The efficacy of targeted therapy, a personalized medicine based on the molecular features of each tumor, is highly affected by the onset of primary or secondary resistance [1,2,3,4,5,6]. Dealing with tyrosine kinases, drug resistance is the main issue to overcome when proposing a targeted therapy approach to patients. Common strategies to overcome drug resistance are chosen according to the mechanism of resistance: drug dose increase, shift to second/third generation inhibitors, or moving to a standard chemotherapy or other treatments. In the past few years, some groups observed in pre-clinical models that resistance to tyrosine kinase inhibitors may also be associated with the development of drug addiction, meaning that upon drug withdrawal, resistant cells die. This phenomenon has been observed in vitro or in vivo both in solid and in hematological disease models: BCR-ABL+ Chronic Myeloid Leukemia (CML) [7], NPM-ALK+ Anaplastic Large Cell Lymphoma (ALCL) [8,9], Gastric Cancer [10], Melanoma [11,12], Lung Cancer [13]. All these models have two features in common: the tumor is driven by a single, potent oncogene necessary to the tumorigenic process and resistance arises from oncogene overexpression. Hence, it is likely that the drug acts as a “molecular brake”, allowing an amount of oncogene driven signaling that is optimal for tumor cells survival and uncontrolled proliferation. If it is too much or too less, the neoplastic cell is over-stressed and dies, as shown by the typical “bell shaped” instead of the classical sigmoidal curve in dose–response tests [8,11]. Too much drug, consistently with the “oncogene addiction” theory, causes apoptosis because the oncogenic signal is abrogated. But without the drug the excess of signal turns out to be toxic, according to the “drug addiction” theory. Interestingly, when drug addicted cells readapt to the absence of the drug, they re-sensitize, at least in part, to the same amount of drug previously stopped [8]. It is likely that a subset of patients relapsing on TKI therapy because of oncogene overexpression, one of the main causes of relapse, may benefit from drug interruption more than from drug increase or drug change. This option would be not only effective, but also safe, since side effects are lacking, and with full compliance. Very little is known about the molecular mechanisms driving drug addiction. Extracellular signal regulated kinase (ERK) pathway signaling seems to be involved in drug addiction associated with drug resistance, and cell death is probably due to genotoxic stress, as highlighted by histone H2AX phosphorylation [8,14]. This result may be paradoxical, since ERK is a well-known apoptosis suppressor, but in some cases, depending upon cell line and type of stimuli, it may act as a pro-apoptotic factor [15,16]. Recently, it has been shown that ERK2 rather than ERK1 has a role in drug addiction related cell death [17]. Moreover, pharmacological suppression of the ERK target mTOR decreases mitochondria oxygen consumption and increases lactate acid production, thus promoting glycolysis and suppressing the Krebs cycle [18]. In NPM-ALK+ ALCL, signal transducer and activator of transcription 3 (STAT3) is one of the main oncogene signaling players. In different cell lines, a fraction of the STAT3 pool has been found in the mitochondrial fraction, where it seems to promote mitochondria activation and enhance ATP production. Paradoxically, it seems to reduce the ROS impact on cells, thus protecting them from oxidative damage [19]. The reasons why STAT3 should increase ATP but not Reactive Oxigen Species (ROS) levels are still unknown. In our work, for the first time, we highlight a mechanism promoting cell death upon drug withdrawal in two drug resistant/drug addicted cell lines from two different hematological malignancies, CML and ALCL, selected as resistant to imatinib or lorlatinib because of oncogene overexpression and, in the ALCL model, also kinase domain mutations. In our models, an excess of oncogenic signaling is related to mitochondria hyper-activation, ROS amount increase, genotoxic stress, and cell death. We also simulated a drug holiday schedule versus a continuous therapy, and we demonstrated that drug holiday significantly delays the onset of drug resistance. When point mutations are co-existing with oncogene overexpression, drug holiday efficacy is lost sooner. By comparing these two models, our data provide a deeper insight into this poorly known mechanism and explore advantages and limits of drug holiday therapy.

## 2. Results

### 2.1. Drug Holiday Schedule Delays the Growth of Drug Addicted Cell Lines

**Figure 1:**
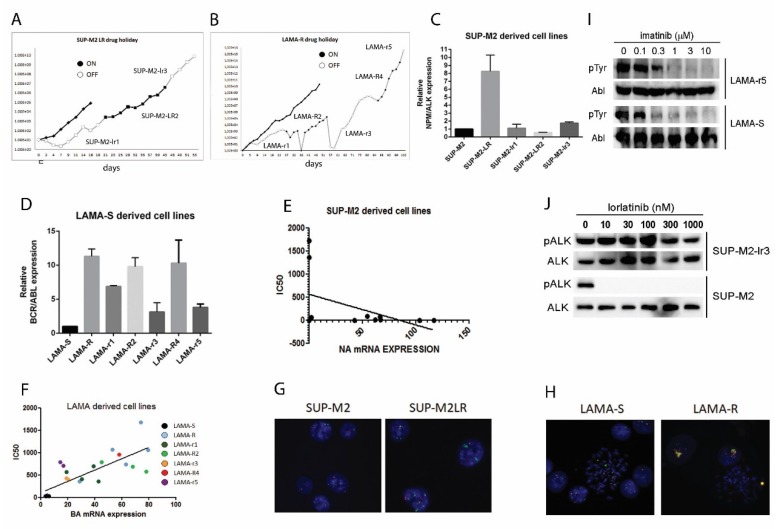
Drug holiday schedule is an effective strategy to keep tumor growth under control in vitro. (**A**) SUP-M2-LR cells or (**B**) LAMA-R cells were kept in culture in the absence of drug. When cells started growing again, specific drug was re-introduced in the medium at the same dose they were used to live before. Again, as soon as new selection occurs, drug was removed again and so on, until no drop in cell count or viability was observed. At each step a new cell line was established and characterized. White dots: cell count in the absence of drug. Black dots: cell count in the presence of drug. (**C**) NPM-ALK expression in SUP-M2 derived cell lines or (**D**) BCR-ABL mRNA expression in LAMA-S derived cell lines. Values of oncogene expression normalized on GUS expression (∆Ct method) are shown. Each dot represents a single PCR reaction normalized on the average of GUS expression values detected in the same experiment. (**E**) correlation between IC50 value and oncogene transcription level in SUP-M2 derived cell lines or (**F**) LAMA derived cell lines. (**G**) Panels representative of the interphase FISH analysis on SUP-M2LR compared to SUP-M2 cell lines or (**H**) LAMA-R compared to LAMA-S. Magnification: 63×. (**I**) LAMA-r5 or (**J**) SUP-M2-lr3 cell lines were challenged with the indicated doses of inhibitor. Corresponding oncogene activity was assessed by western blot.

In order to test the efficacy of a drug holiday schedule, we subcultured LAMA-R (imatinib resistant, always kept in culture at 1 µM imatinib) and SUP-M2-LR (Lorlatinib resistant, always kept in culture at 1 µM lorlatinib) in the absence of the drug. Upon drug withdrawal we observed a first drop in cell number and viability in both cell lines (Figure 1A,B, Figure S1). Within 10 days, both cell lines started to grow again in the medium without drug. Likely a selection had occurred, therefore we established two new cell lines, called LAMA-r1 and SUP-M2-lr1, respectively. We then re-introduced the drug again in the medium and surprisingly a new drop in cell growth and viability was observed, indicating a new selection process. We therefore established two new cell lines, named LAMA-R2 and SUP-M2-LR2. We were able to repeat these on/off rounds five times in LAMA-R and three times for SUP-M2-LR, establishing and characterizing in total eight new cell lines: LAMA-r1, LAMA-R2, LAMA-r3, LAMA-R4, LAMA-r5; SUP-M2-lr1, SUP-M2-LR2, SUP-M2-lr3. We stopped when no viability drop or delay in cell growth was observed (Figure 1A,B, Figure S1). Cell lines selected in the absence of the drug are indicated in lower case, whereas cell lines selected in the presence of the drug are in capital letters. The number indicates how many on/off changes have been done. For each cell line we evaluated the IC_50_ value for the corresponding drug, imatinib or lorlatinib (Table 1), and the amount of oncogene transcript (Figure 1C,D and Table 1). 

Oncogene overexpression in both cell lines was clearly due to gene amplification, as detected by FISH (Figure 1G,H). No mutations were found in BCR-ABL kinase domain in LAMA and LAMA-derived cell lines, by Sanger or deep sequencing, whereas some point mutations in ALK kinase domain were detected in SUP-M2 derived cell lines: a double C1156F/L1198F in the original SUP-M2-LR cell line, or a triple C1156F/L1198F/D1203N mutant after the second drug challenge (SUP-M2-LR2 and SUP-M2-lr3; Table 2). 

These mutations have been previously described in other ALK inhibitor resistance models and found in lorlatinib resistance patients’ specimens [20,21,22,23]. Interestingly, SUP-M2-lr3 completely lost NPM-ALK overexpression, thus relying only on the newly acquired D1203N mutation. In LAMA derived cell lines, we found a significant correlation between BCR-ABL expression and imatinib resistance (Figure 1F), whereas in SUP-M2 derived cells there was no correlation between NPM-ALK expression and lorlatinib resistance (Figure 1E). Therefore, drug holiday selects cells based on their oncogene expression level. When point mutations are present, this correlation is disrupted, and cells growth is under control for a shorter time. We then challenged the two final non-addicted cell lines, LAMA-r5 and SUP-M2-lr3 with alternative TKIs. For LAMA-r5 we chose the most potent ABL inhibitors dasatinib and ponatinib, whereas for SUP-M2-lr3 we chose alectinib, based on the mutations sensitivity profile previously detected [21]. Drug doses were calculated as the 20% of the Baf/3 parental cells IC_50_ [21,24]. Dasatinib and Ponatinib were effective in inducing massive cell death. On the other hand, Alectinib at 90 nM was not able to affect cell growth, in fact after 18 days of treatment no changes in cell growth have been detected (Figure S2). Consistent with the absence of ABL mutations, BCR-ABL activity was completely abrogated in LAMA-r5 treated with 1µM imatinib, suggesting that an ABL independent mechanism of resistance was selected (Figure 1I). On the contrary, in SUP-M2-lr3 ALK phosphorylation is maintained even at high doses, as predicted by mutational status (Figure 1J).

### 2.2. Drug Withdrawal in Drug Addicted Cell Lines Enhances Oncogene Driven Signaling

**Figure 2:**
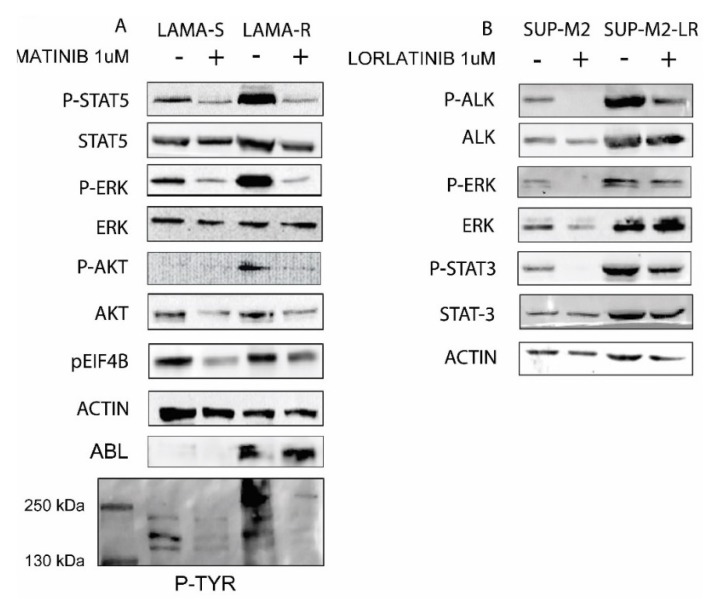
Drug withdrawal causes an oncogene driven signaling overflow. (**A**) LAMA-S and LAMA-R or (**B**) SUP-M2 and SUP-M2-LR cell lines were kept in the presence or in the absence of IMATINIB 1 µM or LORLATINIB 1 µM respectively for 4 h. Then cells were harvested, washed, and lysed. Western blot was performed with the indicated antibodies. + and − indicate the presence or the absence of the drug. + and − indicate the presence or the absence of the drug.

In order to evaluate the molecular effects of drug withdrawal in drug addicted cells, we checked for the activation status of the main oncogene downstream pathways. After a washout of 4 h we checked for the known BCR-ABL targets ERK, STAT5, and AKT activation in LAMA cells, and the NPM-ALK targets ERK and STAT3 in SUP-M2 cells. Activation was evaluated by phosphorylation at key residues. After only 4 h from drug withdrawal all signaling pathways were strongly hyper-activated (Figure 2A,B), whereas as expected, in sensitive cell lines they were shut down by the drugs. In LAMA-R cells, global phospho-tyrosine signal is also higher in the absence than in the presence of imatinib, confirming previous data [7]. It is unlikely this could be due to transcript modulation since cells have been kept in the absence of drug for a short time. Protein stabilization/impaired degradation cannot be excluded. Collectively these findings confirm the hypothesis of the drug as a “molecular brake” in oncogene overexpressing cell lines.

### 2.3. Enhanced Oncogenic Signaling Increases Mitochondria Activity and ROS Production, Inducing Genotoxic Stress

**Figure 3:**
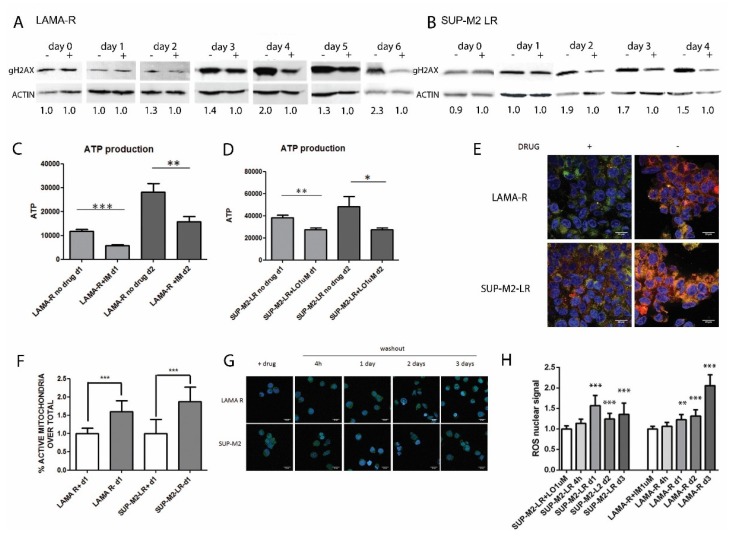
Drug withdrawal in drug addicted cells enhance mithocondria activity and reactive oxygen species (ROS) production, ending up in genotoxic stress. (**A**) The genotoxic marker γ H2AX was detected in LAMA-R and (**B**) SUP-M2-LR at the indicated time points upon drug withdrawal respectively. β –ACTIN was used as loading control. For each panel at least two independent experiments were performed. Lorlatinib and Imatinib were always added to the medium at 1µM. Densitometric analysis normalized on the ON DRUG condition is shown (**C**) ATP production in LAMA-R cell lines or (**D**) SUP-M2-LR was evaluated 1 or 2 days upon drug withdrawal. (**E**) Mithocondria activation increases upon drug withdrawal. The drug, imatinib for LAMA-R and lorlatinib for SUP-M2-LR cell lines, was removed from medium and after 24 h cells were harvested, labelled with Mitotracker and detected at confocal microscopy. BLUE = DAPI. GREEN = total mithocondria. RED = active mithocondria fraction. Scale bar: 20 µM. A representative panel is shown (**F**) The histogram represents the average of two independent experiment each cell line with Mitotracker. (**G**) ROS production is increased after 1 to 3 days upon drug withdrawal. The drug was removed from medium and after 4 h, 1 day, 2 days or 3 days. Cells were stained with CellROX® Green Reagent and signal was detected with confocal microscopy. Scale bar: 20 µM. A representative panel is shown. (**H**) The histogram represents the average of two independent experiments performed with CellROX® Green Reagent. * *p* < 0.05, ** *p* between 0.05 and 0.01 *** *p* < 0.01. + and − indicate the presence or the absence of the drug.

We know that in drug addicted cells, drug withdrawal is accompanied by genotoxic stress [8]. We confirmed in our cell lines that after four days for LAMA-R and two days for SUP-M2-LR there is an increase in γH2AX (Figure 3A,B). Since this could be related to mitochondria activity, we first evaluated ATP production and mitochondria activity in drug-addicted cells upon drug withdrawal. We found after two days a significant increase in ATP production in both drug addicted cell lines. In both cell lines ATP production is significant since one day from drug removal (Figure 3C,D). ATP production is impaired in treated parental cells, consistently with drug induced toxicity (Figure S3). Confocal microscopy experiments revealed that ATP increase correlated with a significant enhancement in mitochondria activity since one day of drug withdrawals (Figure 3E,F). Since mitochondria are an important source of reactive oxygen species (ROS) in mammalian cells, we evaluated ROS production using fluorescent probes and we found a significant increase in ROS production, with a peak at day 1 for SUP-M2-LR cell lines and at day 3 for LAMA-R (Figure 3G,H). Notably, cells have a heterogeneous release of ROS (Figure 3G). This is probably linked to the amount of oncogene-driven signaling present upon drug withdrawal and may explain the inability to completely defeat the tumor cells with a drug holiday schedule (Figure 1A,B). These results indicate that drug withdrawal is followed by increased ROS production by mitochondria, leading to DNA damage, in drug-addicted cells.

### 2.4. Enhanced Oncogenic Signaling Causes Cell Death

**Figure 4:**
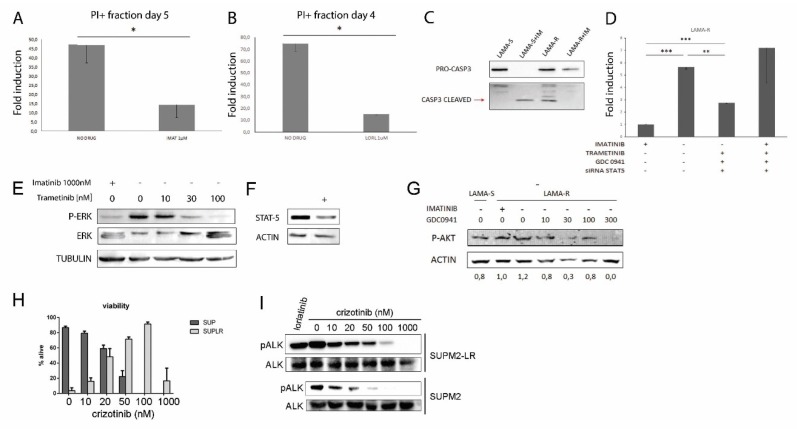
Drug addicted cells die upon drug withdrawal. Simultaneous inhibition of three downstream pathways partially rescue drug addiction-induced cell death. (**A**) PI+ fraction is detected on LAMA-R cells 5 days upon imatinib withdrawal. (**B**) PI+ fraction is detected on SUP-M2-LR cells 4 days upon lorlatinib withdrawal. (**C**) Cleaved caspase 3 was detected five days upon drug withdrawal in LAMA-S and LAMA-R. (**D**) Cells were treated with the indicated drugs at doses: IMATINIB (ABL inhibitor), 1 µM. TRAMETINIB (MEK inhibitor), 30 nM. GDC0910 (PI3K inhibitor), 100 nM. siRNA STAT5, 50 nM. LAMA-R cells were transfected or not with STAT-5 siRNA in the presence of imatinib, then after 24 h they were challenged with one or a combination of inhibitors. Five days after drug withdrawal cells were collected and the PI+ fraction was evaluated by flow cytometry. The histogram indicates the average of at least two independent experiments (**E**,**F**,**G**) The efficiency of target inhibition in LAMA-R cells was detected by western blot after 4h (panels E and G) or 24 h (panel F). (**H**) 1 h after lorlatinib withdrawal, cells were treated with the indicated crizotinib doses. Cell viability was assessed by trypan blue count after four days. (**I**) The efficiency of target inhibition in SUP-M2-LR cells was detected by western blot 4 h after crizotinib treatment. * *p* < 0.05, ** *p* between 0.05 and 0.01 *** *p* < 0.01. + and − indicate the presence or the absence of the drug.

To evaluate the biological consequences of enhanced signaling and ROS levels, cell death was assessed. Drug withdrawal caused a significant increase in cell death, defined as the PI+ cells fraction, in both LAMA-R and SUP-M2-LR cell lines (Figure 4A,B). Interestingly, cell death timing was different: in LAMA-R cells a peak in PI+ fraction was seen five days after drug withdrawal, whereas in SUP-M2-LR cell death occurred in 3–4 days, so for this line drug withdrawal-induced cell death kinetics is shorter. At the same time points, there is an increase in the fraction of late apoptotic cells, defined as AnnexinV+/PI+, although this difference was not statistically significant (Figure S4). This let us hypothesize that apoptosis is co-existing with other mechanisms of cell death. Caspase 3 cleavage in LAMA cells further confirmed drug withdrawal induced cell death (Figure 4C). To confirm that death is a consequence of an excess of oncogenic driven signaling, we performed a rescue experiment, by partial inhibition of the three main BCR-ABL driven downstream pathways (STAT5, ERK, and PI3K), while removing imatinib from the medium. Cell death was evaluated six days later (Figure 4D). We used a MEK inhibitor, trametinib (Figure 4E), a siRNA directed against STAT-5 (Figure 4F) and a PI3K inhibitor, GDC-0941 (Figure 4G). As expected, imatinib withdrawal increased significantly the amount of PI+ cells, and the inhibition of all downstream pathways in the presence of imatinib further increased the amount of dead cells. However, the simultaneous partial block of the three downstream pathways was able to rescue cells from imatinib withdrawal induced cell death, indicating that toxicity is indeed due to an excess of oncogenic signaling. Conversely, it was not possible to perform the same rescue experiment in SUP-M2-LR cell line. An effective and non-toxic STAT3 downregulation was very difficult to obtain, both by siRNA technique or by pharmacological inhibition. Moreover, when STAT3 inhibition did not lead to massive cell death, the only effective variable in inducing cell survival or cell death was the presence of lorlatinib in the medium (Figure S5A,B). Although ERK is known to be one of the key players of drug addiction induced cell death, single ERK inhibition by trametinib could not rescue cell death (Figure S5C,D). For this reason, we rescued drug withdrawal induced cell death by adding several doses of a different ALK inhibitor, crizotinib (Figure 4H). In SUP-M2 cell lines, crizotinib IC_50_ is about 56 nM [25]. Crizotinib was effectively able to rescue cell death induced by lorlatinib withdrawal, and rescue capability correlated with the amount of ALK inhibition. Interestingly, at high crizotinib doses (1000 nM), cell viability dramatically dropped, in line with the bell-shape type of response. This result confirms that ALK-dependent signals trigger cell death when lorlatinib is withdrawn.

Finally, since drug withdrawal toxicity is mediated by ROS enhanced released, we used reduced glutathione (GSH) to restore cell death upon drug withdrawal. In both LAMA-R and SUP-M2LR cell lines there is a mild improvement in cell viability upon GSH treatment, especially at GSH 100 nM and 20 nM respectively. This trend was correlated with a decreased ROS amount detected at confocal microscopy (Figure S6).

### 2.5. Excess of ProliferAtive Stimuli and ROS Production Induces Stress Response Activation and Oncogene Effectors Downregulation

**Figure 5:**
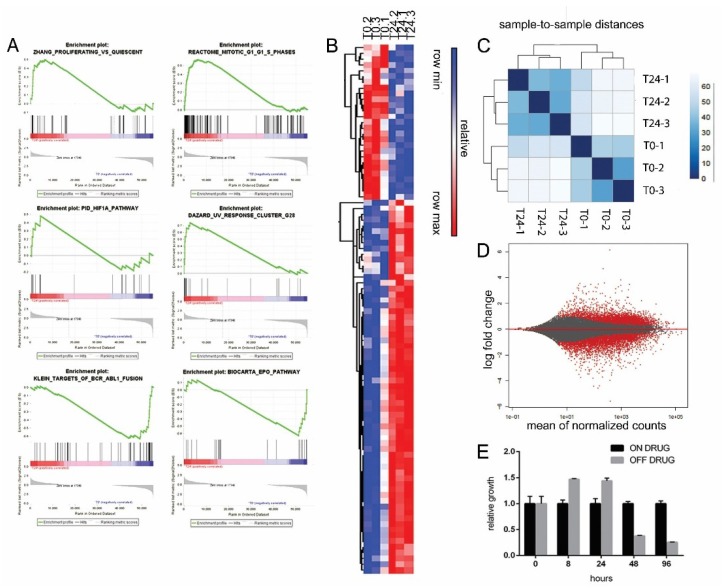
RNA sequencing was performed on LAMA-R cells, before and 24 h after drug withdrawal. Three independent experiments were analyzed. (**A**) Gene Sets Enrichment Analysis (GSEA). Six representative gene sets are shown. (**B**) heat map related to panel A gene sets. (**C**) Sample-to-sample distance matrix of Pearson correlation coefficients highlighting the high similarity of the three replicates. (**D**) MA plot showing the fold change as a function of average gene expression, before and after drug withdrawal. Differentially regulated genes reaching statistical significance are highlighted in red. (**E**) LAMA-R cells growth was assessed at the indicated time points after imatinib withdrawal (grey bars) and normalized on cells kept continuously on drug (black bars).

**Figure 6:**
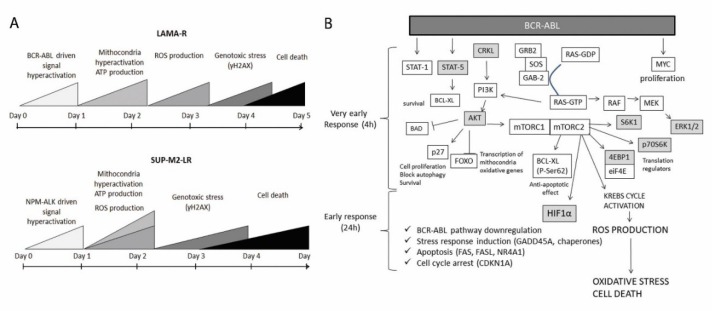
Representation of the model proposed. (**A**) Diagram comparing the temporal sequence of events leading to cell death in LAMA-R and in SUP-M2-LR cell lines. (**B**) Scheme representing the pathways activated upon drug withdrawal in LAMA-R. Dark boxes indicates the aforementioned genes.

To further understand the transcriptional changes occurring upon drug withdrawal, we collected RNA from LAMA-R, before and 24 h after drug withdrawal. Gene Sets Enrichment Analysis (GSEA) from RNA sequencing data highlighted a significant upregulation of genes involved in proliferation, ROS response, apoptosis, stress response, cell cycle arrest and a significant downregulation in gene sets comprising BCR-ABL downstream signaling effectors, such as JAK2 and STAT5 (Figure 5A,B). Transcriptional profile changes upon drug withdrawal are profound and quite homogeneous among replicates as shown by sample-to-sample Pearson correlation analysis (Figure 5C), and differential gene expression analysis showing a high number of dysregulated genes (Figure 5D). All these data support the previous findings, where an excess of oncogene driven signaling promotes uncontrolled early cell proliferation (Figure 2C), and increases ROS production (Figure 3G,H). As a consequence, stress response (e.g., GADD45A, chaperones), cell cycle arrest (CDKN1A), and apoptosis (Fas, FasL, NR4A1) -related genes were upregulated while BCR-ABL effectors were downregulated. Figure 6A shows the temporal sequence of the events occurring after drug withdrawal. Figure 6B summarizes the pathway involved, according to the model proposed.

## 3. Discussion

In the last few years several studies linked the concept of drug resistance to drug dependency. In both hematological and solid tumors driven by a single, strong oncogene, when drug resistance is due to oncogene overexpression, drug removal from the medium is sufficient to cause massive cell death. Likely, in case of TKI relapse due to oncogene increased expression, a new, efficient therapeutic option would be treatment suspension or a drug holiday schedule, with three important advantages: first, drug suspension should not cause any side effect. Second, patients’ compliance should approach 100%, since everybody would remember not to take a drug. Third, it is a consistent money saving from the healthcare system. Clinically there are a few anecdotal examples of melanoma cases treated with a drug holiday schedule, demonstrating that it is not only feasible, but also effective [14,26]. In our work for the first time we highlight the mechanisms leading to cell death upon drug withdrawal in two hematological cell lines drug resistant/drug addicted: one, LAMA-R, from a CML BCR-ABL+ imatinib resistant model, the second, SUP-M2-LR, from an ALCL NPM-ALK+ lorlatinib resistant model. LAMA-R model is limited, since it had been established from a blast crisis patient whereas, thanks to TKI treatment, actually only a small fraction of patients (1–1.5%) progress to accelerated phase and blast crisis [27]. Despite this observation, LAMA cell lines, as well as the other blast crisis CML derived cell lines, have been recognized and broadly used as a simple, good model for this disease in a great amount of studies. In LAMA-R, no mutations in BCR-ABL kinase domain were found as a co-cause of drug resistance. On the opposite, in SUP-M2-LR, ALK kinase domain point mutations were detected (C1156F+L1198F, or C1156F+L1198F+D1203N). These mutations have already been found to confer resistance to small ALK inhibitors [20,21,23]. In particular, several works identify D1203N as a lorlatinib resistance contributing mutation, both in vitro and ex vivo [21,22,28]. Likely the presence of point mutations shortened the on/off rounds number, since natural selection pushed toward a population not sensitive to drug holiday, the mutated one. Both cell lines overexpressed the oncogene: BCR-ABL transcript was 11.5 times more abundant, clearly due to gene amplification (Figure 1D,G). NPM-ALK transcript was more than 100 times higher compared to sensitive cell lines (Table 1), and this was again a consequence of oncogene amplification (Figure 1H). In both cell lines, a few hours upon drug withdrawal, a strong enhancement in oncogene-driven signaling happens, followed by mitochondria hyper-activation, increase in ATP production, and a boost in ROS production and γH2AX, a marker of genotoxic stress. Cell death occurs after a couple of days. The same process was observed in both cell lines, with different timing (Figure 6A). This might be due to a difference in oncogene effectors. AnnexinV-PI staining revealed that a fraction of cells never expressed AnnexinV on their surface so probably more than one cell death pathway was activated. Despite cell death, in both cell lines after drug withdrawal a population able to live without drug was selected (Figure 1A,B). The in vitro simulation of drug holiday schedule delayed significantly tumor growth compared to cells kept continuously in the presence of the drug. (102 days for LAMA-E and 43 days for SUP-M2LR). Then, we could no more induce a drop in cell population by removing drug from the medium. There is a significant correlation between drug resistance and oncogene expression in LAMA-R, whereas no correlation was found in SUP-M2-LR (Figure 1E,F). According to the “molecular brake” hypothesis, drug withdrawal is counter-selecting only cells expressing the highest amount of oncogene and positively selecting the ones with a mild overexpression, that are also less drug addicted. Reintroducing the drug, cells expressing the highest oncogene levels will survive, and so on for a couple of rounds. On the opposite, in the presence of point mutations, drug reintroduction will foster mutated cells, independently from oncogene expression level. In this case shifting to other small molecules may be a successful therapeutic option, if they are active on the mutant. The heterogeneity observed in ROS production is probably the reason why we just delayed and did not completely defeat tumor cells with the drug-holiday approach. Assuming that neoplastic cells arise randomly and continuously accumulate genetic aberrations, our polyclonal cell lines recapitulate possible changes due to selective pressure. Rescue experiments on LAMA-R cells confirmed a cause–effect relationship between oncogene signaling excess and cell death. The rescue is only partial, but this may be because BCR-ABL driven signaling involves more than the three pathways, so also signaling shut down is incomplete. Unfortunately, we could not replicate the rescue in SUP-M2-LR cells, likely because STAT3 signaling is crucial for NPM-ALK addicted cell lines and it was not possible to finely tweak its activity without causing catastrophic effects, and/or because NPM-ALK acts on a broader number of effectors, making it impossible to see a result by abrogating just three downstream pathways. In this case the only effective option to induce cell death was to abrogate ALK signaling. Re-introduction of a different ALK inhibitor upon lorlatinib withdrawal effectively rescued off drug-induced cell death in a dose-dependent manner (Figure 4H,I), demonstrating that cell death depends upon oncogene activity modulation. At high doses, cell viability drops dramatically, according to the concept that cell survival is guaranteed by the right amount of oncogene driven signaling, recapitulating the well-known dose–response bell-shaped curve profile. [8]. A mild rescue has been obtained upon reduced glutathione treatment, an effective way to block the oxidizing ROS effect. Probably glutathione effect was mild because of the instability of this compound (Figure S5). The two different models of drug addiction presented are not recapitulating exacting the same molecular mechanisms, likely because of slight differences in the oncogene-driven downstream pathways. However, in both models drug withdrawal induced mitochondria hyperactivation, ROS release, and massive cell death. Cell death induced by drug withdrawal was observed by several groups in different cancer models [9,10,11,12,13,17] RNA sequencing of LAMA-R before and 24 h after drug withdrawal highlighted profound transcriptional changes occurring upon drug withdrawal. We observed a strong and redundant downregulation in genes downstream the BCR-ABL pathway, that may be a reaction to an uncontrolled BCR-ABL pathway hyperactivation. Many genes involved in cell proliferation and mitogenesis were upregulated, probably because of extreme BCR-ABL upregulation, whereas others involved in cell cycle arrest were upregulated, in a conflictual response to this uncontrolled proliferation stimulus. Indeed, at early hours after imatinib removal, cells showed enhanced proliferation (Figure 5E). At 24 h upon drug withdrawal a small but significant increase in ROS production was seen (Figure 3H); consistently the HIF1a gene set, known to be upregulated upon ROS release, was enriched. At the same time, a dramatic change in transcriptome was observed (Figure 5). Data indicate a strong downregulation of BCR-ABL driven genes and an increase in stress response related genes, like chaperones, apoptosis related genes (Fas, FasL, NR4A1), and both genes promoting cellular proliferation and cell cycle arrest. These conflictual results can be explained as a reaction to the uncontrolled proliferation stimulus driven by the sudden overload of oncogene signaling due to drug withdrawal. On one side BCR-ABL drives a pro-mitotic signal and induces ROS. On the other side the cell is reacting to ROS or to the catastrophic pro-mitotic signal, or both. In conclusion, for the first time we describe the mechanism underlying drug addiction-related cell death and we compared two different hematological diseases with or without the simultaneous presence of kinase domain point mutations (Figure 6A,B). Checking carefully the presence of point mutations as a co-cause of resistance would be necessary to predict the long-term effectiveness of a drug holiday schedule. In LAMA and SUP-M2 model drug holiday was efficient in keeping cell growth under control more than inducing a massive cell death, whereas in our previous work [8] two cell lines out of three never grew again upon brigatinib withdrawal. According to our model, heterogeneity of the disease may play a role in drug holiday efficacy. Still we do not know the threshold of oncogene overexpression required to shift from drug resistance to drug addiction, neither if this threshold is disease specific or not, so actually it is difficult to predict if a patient will benefit or not from drug suspension. Further work should be made in order to optimize drug addiction prediction on patients’ samples. These findings provide a deeper knowledge of this phenomenon, useful for a future translation into clinic.

## 4. Materials and Methods 

### 4.1. Cell Lines, Drugs and RNA

LAMA-S and LAMA-R (imatinib resistant) were kindly provided by dr Junia Melo and selected as previously described [7]. Cells were cultured in RPMI-1640 supplemented with 9% FBS U.S. origin (OPTICLONE serum, Euroclone—Pero, Italy), 100 U/mL penicillin, 100 mg/mL streptomycin, and 2 mmol/L L-glutamine (Invitrogen, Carlsbad, CA, USA), in the presence of 1 µmol/L imatinib and incubated at 37 °C with 5% CO_2_. Medium was replaced every 48–72 h with fresh imatinib. SUP-M2 cell line was purchased from DSMZ, where they are routinely verified using genotypic and phenotypic testing to confirm their identity. SUP-M2-LR (lorlatinib resistant, also referred to as SUPM2-LR1000, [23]) were obtained by subculturing SUP-M2 cell line with increasing lorlatinib doses. Cells were kept in culture using 9% Euroclone USDA serum supplemented with 1 µmol/L lorlatinib. Medium was replaced every 48–72 h. To evaluate cumulative cell growth, the total amount of cells was multiplied by the product of all previous dilution factors. Lorlatinib was kindly provided by Pfizer. Imatinib mesylate was synthesized as described [29]. Trametinib, GDC-0941, BP-1-102 were purchased by SelleckChem (Munich, Germany). For STAT-5 and STAT-3 silencing, specific siRNAs were purchased from Cell Signaling Technology—Danvers, MA, USA (#6275 and #6580) and used at 50 nM concentration in medium alone and 20 µL of INTERFERIN (Polyplus transfection, -Bioparc—850, Bd S. Brant—67400, Illkirch, France). Reduced Glutathione (GSH) was purchased from Sigma-Aldrich (Saint Louis, MO, USA).

### 4.2. Q-RT PCR and Sequencing

Three to five million cells were lysed in Trizol® (Invitrogen, Carlsbad, CA, USA) and RNA was extracted following manufacturer instructions. 2 µg of total RNA was retrotranscribed in a 50 µL reaction using Reverse Transcription Reagents (Applied Biosystems-Life Technologies, Carlsbad, CA, USA). Quantitative Real time PCR (Q-RT PCR) was performed on a Stratagene-MX3005P (Agilent technologies, Santa Clara, CA, USA) under standard conditions. BCR-ABL amplification was obtained by RT-qPCR using TaqMan® Brilliant II QPCR Master Mix (Agilent technologies), whereas NPM-ALK amplification was performed by SybrGreen assay using Brilliant III Ultra-fast SYBR Green QPCR Master Mix (Agilent technologies), according to manufacturer instructions. NPM-ALK primers were previously described [20]; BCR-ABL primers used are BCR_Ex13FW (5′ TCCGCTGACCATCAAYAAGGA 3′), ABL_Ex2REV (CACTCAGACCCTGAGGCTCAA), the probe was ALB_Ex2REV (CCCTTCAGCGGCAGTAGCATCTG). Housekeeping genes were GAPDH for SybrGreen assay [20] and GUS for TaqMan assay [30]. PCR was performed using Q5 high fidelity polymerase (New England Bioscience) and a previously described pair of primers specifically designed for NPM-ALK [20] or BCR-ABL: BCRb2c_intFW and Abl4Rev [24]. PCR products were sent to GATC biotech for Sanger sequencing. For deep sequencing, after RNA extraction from Trizol® solution and retrotranscription, BCR-ABL kinase domain was amplified using the following primers: BCR b2c int fw: 5′GTGAAACTCCAGACTGTCCA 3′ and Abl4Rev: 5′CTTCTCTAGCAGCTCATACAC 3′. Amplification product was run on agarose gel, purified and sent to GalSeq S.r.l. for deep sequencing. The fragments were sequenced at 10,000 × coverage in paired-end mode (2 × 150 bp). Fastq files were aligned to the reference amplicon sequence and variants annotated and filtered against control (parental cell line). For RNA-sequencing, three independent LAMA-R cultures were cultured in the absence of imatinib. Total RNA was extracted with TRizol before (controls) and 24 h after withdrawal. RNA libraries were prepared, sequenced at a depth of 15 million clusters per sample and analyzed, as described [31].

### 4.3. Western Blot

Cells were plated in the presence or in the absence of the indicated drug for a minimum of 2 h to a maximum of 5 days. Then, 10^6^ or 1.5 × 10^6^ cells were harvested, washed in PBS at 4 °C and lysed in 100 µl of lysis buffer supplemented with protease inhibitor cocktail [25]. For γH2AX detection, after washing, cells were lysed directly in Laemmli buffer supplemented with 10% β-mercapto-ethanol at working concentration. Before loading on SDS-PAGE, lysates were denatured for 10 min at 97 °C. Equal volumes or 20–100 µg of proteins were loaded and transferred to nitrocellulose (Hybond ECL, Amersham, Little Chalfont, UK), and incubated overnight at 4 °C or 1 h at room temperature with primary antibody. Following at least 1 h of incubation with secondary horseradish peroxidase–conjugated antibody (anti-rabbit or anti-mouse, BioRad, Hercules, CA, USA), chemiluminescence was detected as recommended using Kodak image station 440. The following Cell Signaling Technology antibodies were used: P-STAT5 (#9356), P-ERK (#4370), ERK (#9102), P-AKT (#13038), AKT (#9272), P-EIF4B (#3591), P-STAT3 (#9138), STAT3 (#12640), γH2AX (#9718), CASPASE-3 (#9665). Anti phosphotyrosine is from Sigma (#P4110, (Saint Louis, MO, USA), while anti STAT5 from Santa Cruz Biotechnology, Dallas, TX, USA (#SC-835). The loading controls anti-TUBULIN and anti β-ACTIN were purchased from Abcam, Cambridge, UK (#6046), and Sigma (#A2228) respectively. 

### 4.4. ATP Detection, Apoptosis, MTS Assay and Cell Proliferation Assay

Cells were kept with or without drug for 24 or 36 h. 50,000 cells per well were seeded in triplicate on a 96 well plate and intracellular levels of ATP were detected using ATPlite kit (Perkin Elmer, Waltham, MA, USA). To detect apoptosis, cell lines cultured with or without the indicated drug for the indicated time (from 3 to 7 days), then washed with PBS at 4 °C, resuspended in Annexin binding buffer and stained with AnnexinV-/Propidium Iodide kit (Bioscience, Franklin Lakes, NJ, USA). Samples were analyzed using a FACScalibur cytofluorimeter. IC_50_ values were calculated plating 10^4^ cells per well in a 96 well plate in the presence of different drug concentrations. The highest concentration was 10 µM both for imatinib and lorlatinib, followed by serial 1:3 dilutions. In the control raw no drug was added to the medium. After 72 h, 20 µl of CellTiter 96® AQueous One Solution Cell Proliferation Assay (Promega, Madison, WI, USA) were added to the medium. Absorbance was detected at 490 nm. For cell proliferation assay, Cell Proliferation Assay Kit (Fluorometric, BioVision, Milpitas, CA, USA) was used according to instructions. Fluorescence was read using infinite F200PRO (Tecan, Männedorf, Swizerland). 

### 4.5. Fluorescence Imaging and FISH

At the indicated time points after drug withdrawal the cells were stained and imaged for mitochondria activity or ROS accumulation. For mitochondria analysis, samples were incubated for 1 h in standard culture conditions with 50 nM MitoTracker Red CMX and Green (Thermo Fisher, Waltham, MA, USA). Accumulation of red dye depends upon mitochondrial membrane potential, a key indicator of mitochondrial function, while the green signal is proportional to the mitochondria mass. After incubation cells were washed twice with PBS, stained with Hoechst 33342 (Thermo Fisher) and mounted on glass slides with a 90% (v/v) glycerol/PBS solution.

ROS production was assessed by staining cells with CellROX® Green Reagent (ThermoFisher). The probe, upon oxidation by ROS, binds to DNA allowing the quantification of oxidative stress. After staining with CellROX®5 μM for 30 min in culture conditions, cells were fixed for 15 min at room temperature in 4% paraformaldehyde, then washed with phosphate-buffered saline, stained with DAPI (Thermo Fisher) and glass coverslips were mounted on glass slides with a 90% (v/v) glycerol/PBS solution. 

Samples images were acquired with a confocal laser-scanning microscope (Zeiss LSM 710, Jena, Germany) using a 63×, 1,4 N/A oil-immersion objective, applying an additional hardware zoom was required. All the acquisition settings were kept constant for every single experiments. Interphase FISH was performed as previously described [20]. For BCR-ABL on LAMA cells, the BCR/ABL1 plus probe from Metasystem was used

### 4.6. Software, Statistical Analysis and Bioinformatic

IC_50_ values were calculated as the drug concentration that gives half maximal response and was analyzed using GraphPad Prism version 5.0. q-RT PCR data were analyzed using the ΔCt method, normalized on the proper housekeeping gene. Flow cytometry data were analyzed using FCS EXPRESS v.4 software, while Sanger sequencing mutations were detected by Vector NTI advance 10 and Mutant Surveyor software. Densitometry was performed using ImageJ software and normalized on the loading control. MTS assay and ATPlite results were analyzed by Student’s unpaired *t*-test. Fluorescence imaging data semi-quantitative analysis was performed using specifically designed macro with ImageJ software (http://rsbweb.nih.gov/ij/), whereas statistical analysis was performed using one-way ANOVA test or unpaired *t*-test.

## 5. Conclusions

In several tumor models, solid and hematological, drug-addiction related to drug resistance is an emerging paradox. Depending on the mechanism selected, drug resistance may be a double-edged sword, a new weapon developed by cancer cells that clinicians can potentially use against them. For the first time, in this work we described the mechanisms sustaining drug withdrawal related toxicity. Drug withdrawal in drug addicted cells is dramatically changing the transcriptional profile, inducing mitochondria hyper-activation and oxidative stress, while the simultaneous presence of point mutations is affecting drug holiday efficacy. Further studies will be necessary to translate this knowledge into the clinic, nevertheless this knowledge is an important step toward this goal.

## Figures and Tables

**Table 1:** IC50 values, Relative Resistance Index, and oncogene transcript level normalized on the parental cells (Relative Transcript Level) are shown in Table 1.

Cell Line	Drug	IC50 [nM]	RR Index	Relative Transcript Level
LAMA-S	IMATINIB	22	1	1
LAMA-R	IMATINIB	773	35	11
LAMA-r1	IMATINIB	490	22	7
LAMA-R2	IMATINIB	714	32	10
LAMA-r3	IMATINIB	413	19	4
LAMA-R4	IMATINIB	953	43	10
LAMA-r5	IMATINIB	744	34	4
SUP-M2	LORLATINIB	2.13	1	1
SUP-M2LR	LORLATINIB	>3000	>1400	8
SUP-M2lr1	LORLATINIB	67	3	1
SUP-M2LR2	LORLATINIB	3114	141	1
SUP-M2lr3	LORLATINIB	1530	69	2

**Table 2:** mutations detected by Sanger sequencing in SUP-M2 derived cell lines.

Cell Line	Mutations		
SUP-M2	-	-	-
SUP-M2 LR	C1156F	L1198F	-
SUP-M2 lr1	-	-	-
SUP-M2 LR2	C1156F	L1198F	D1203N
SUP-M2 lr3	C1156F	L1198F	D1203N

## Supplementary Materials

The following are available online at http://www.mdpi.com/2072-6694/10/12/509/s1, Figure S1: Viability of LAMA-R and SUP-M2LR during the drug holiday schedule, Figure S2: SUP-M2lr3 and LAMAr5 growth and viability after alternative TKI treatment, Figure S3: ATP production after 24 or 48 h of TKI treatment in LAMA-S and SUP-M2 parental cells, Figure S4: Double positive ANNEXIN V-PI fraction after 5 days (LAMA-R) or 4 days (SUP-M2 LR) from drug withdrawal, Figure S5: Simultaneous inhibition of MEK, PI3K and STAT3 in SUP-M2 LR cells after lorlatinib withdrawal, Figure S6: GSH treatment is able to partally restore cell death after drug withdrawal.
