# Supplementary material for: Mitochondrial Hyperactivation and Enhanced ROS Production are Involved in Toxicity Induced by Oncogenic Kinases Over-Signaling
Source: Cancers (Basel). 2018 Dec 12;10(12):509. doi: 10.3390/cancers10120509 (PMC6316814; doi:10.3390/cancers10120509)
Supplement: Supplementary file 1 [file cancers-10-00509-s001.pdf]

Supplementary Materials: Mitochondrial Hyperactivation and Enhanced ROS Production are Involved in Toxicity Induced by Oncogenic Kinases Over-Signaling.

Monica Ceccon, Mario Mauri, Luca Massimino, Giovanni Giudici, Rocco Piazza, Carlo Gambacorti-Passerini and Luca Mologni

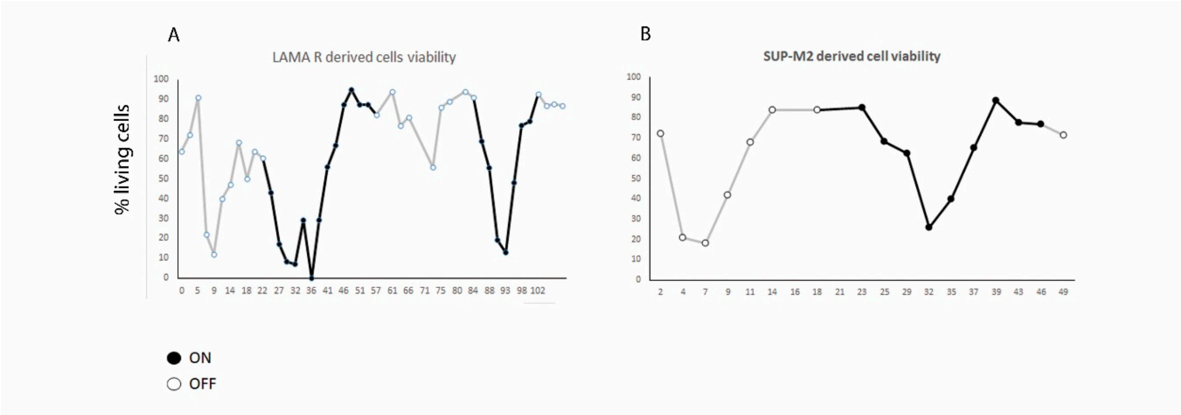

Figure S1. Viability of LAMA-R and SUP-M2LR during the drug holiday schedule. Days of culture are shown on the X axis (**A**) Drug holiday simulation on LAMA-R cells. White dots: drug off. Black dots: drug on. (**B**) Drug holiday simulation on SUP-M2 LR cells. White dots: drug off. Black dots: drug on.

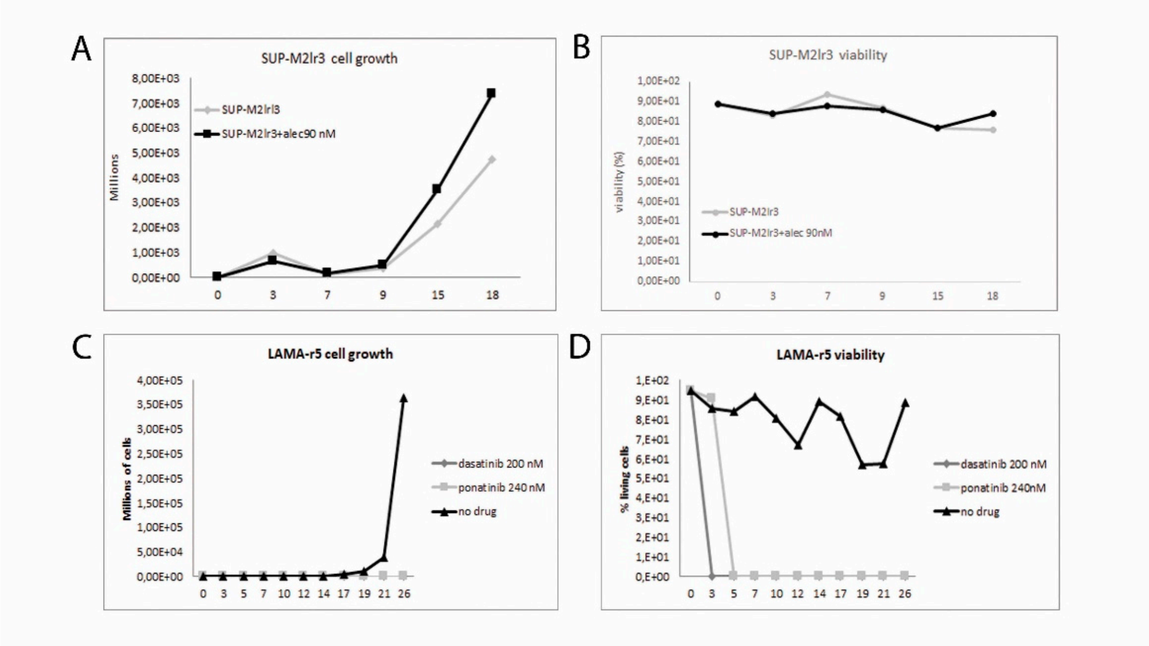

Figure S2. SUP-M2lr3 and LAMAr5 growth and viability after alternative TKI treatment. Days of culture are shown on the x axis. (**A**) SUP-M2 lr3 were grown in the presence of the indicated drug. The number of cells was normalized on the previous dilution factor. (**B**) SUP-M2 lr3 viability was also evaluated. Viable cell count was assessed by trypan blue. (**C**) LAMA-R were grown in the presence

of the indicated drug. The number of cells was normalized on the previous dilution factor. (**D**) LAMA-R viability was also evaluated. Viable cell count was assessed by trypan blue.

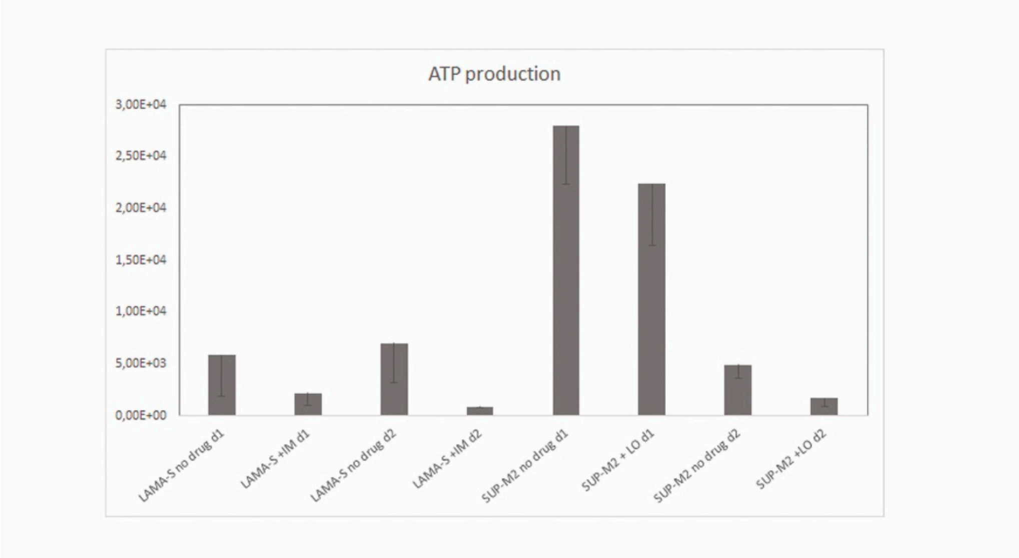

Figure S3. ATP production after 1 or 2 days of TKI treatment in LAMA-S and SUP-M2 parental cells.

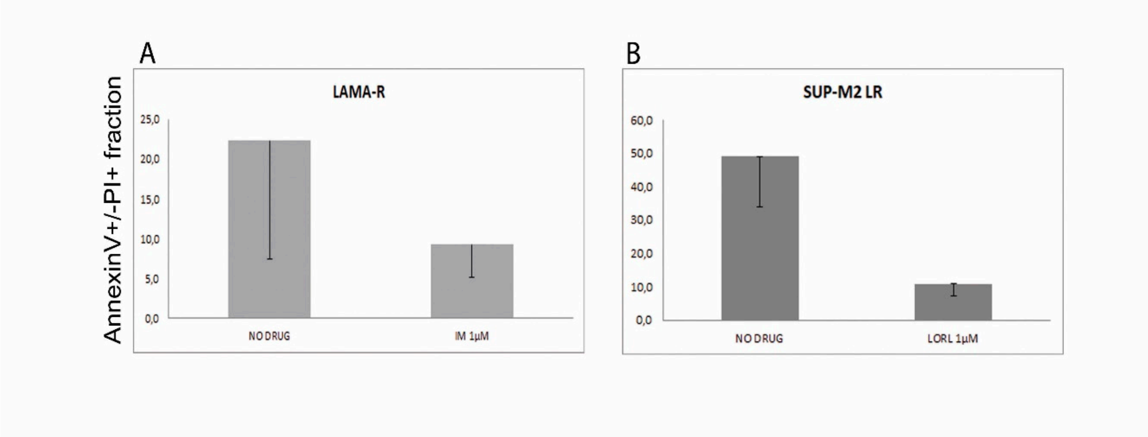

Figure S4. Late apoptotic cells fraction was evaluated at FACS analysis as the double positive ANNEXIN V+ / PI+. (**A**) Late apoptotic LAMA-R cells 5 days after drug withdrawal, (**B**) Late apoptotic SUP-M2 LR cells 4 days after drug withdrawal.

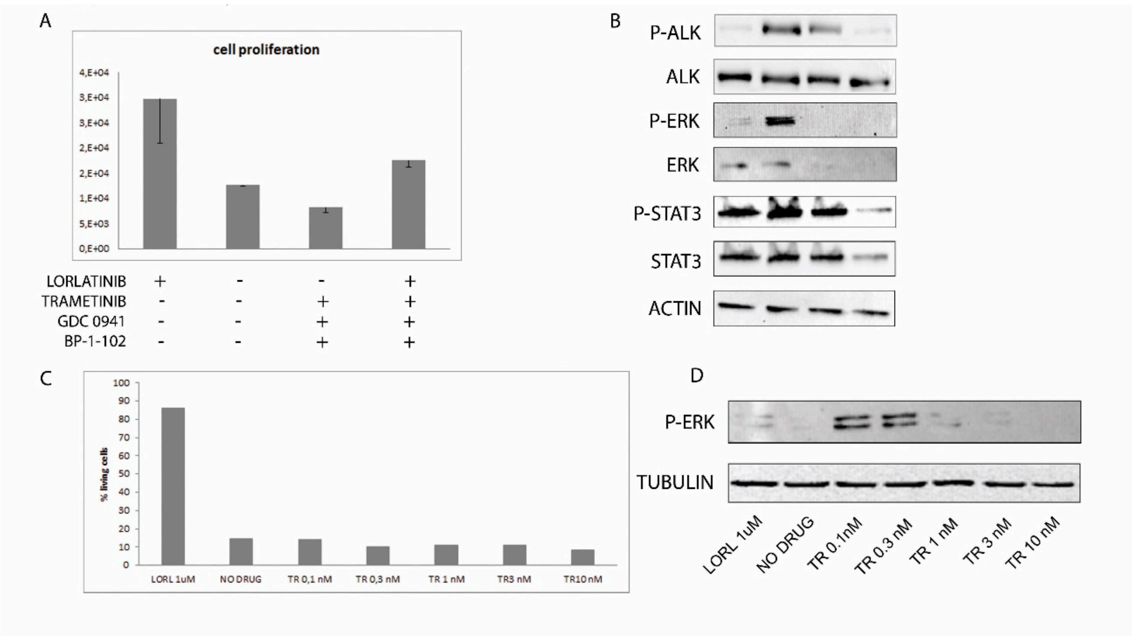

Figure S5. Simultaneous inhibition of MEK, PI3K and STAT3 in SUP-M2 LR cells after lorlatinib withdrawal. (**A**) SUP-M2 LR were treated with the indicated drugs at the following concentrations: Lorlatinib [1 µM], trametinib [3 nM], GDC 0941 [100 nM], BP-1-102 [8 µM]. After 4 days cells were harvested an, equal amount were plated and stained with Florometric Cell Proliferation Assay Kit. (**B**) Indicated targets, where possible, were analyzed by western blot. (**C**) SUP-M2 LR were plated with lorlatinib, without drug or with the single tramentinib at indicated doses. Viability was assessed at trypan blue count. (**D**) ERK activation was assessed for each condition.

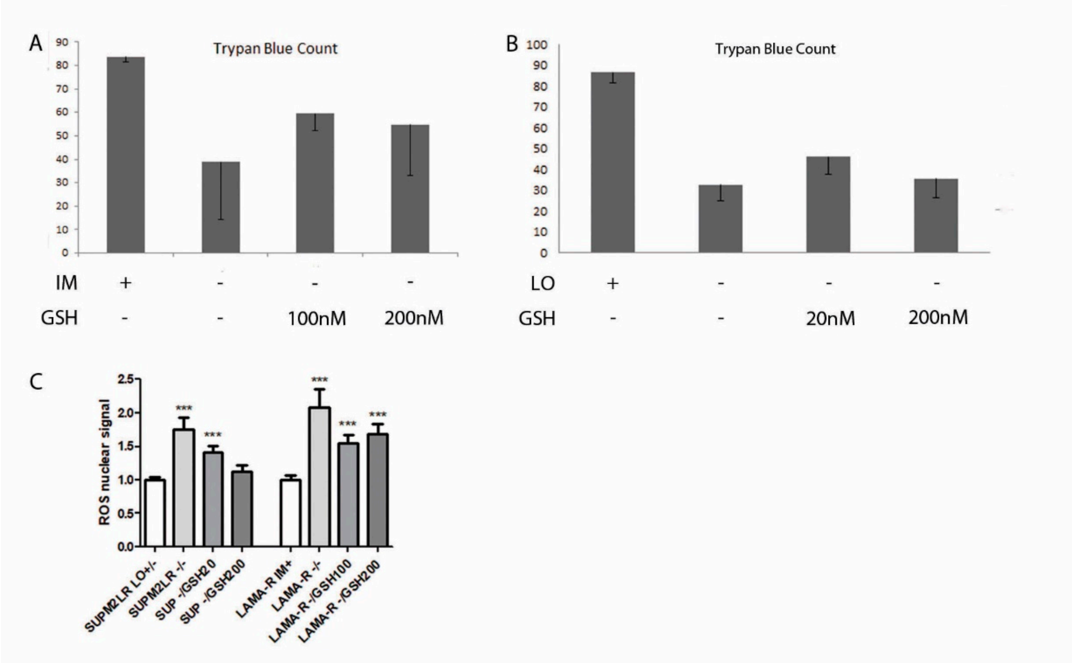

Figure S6. GSH treatment is able to partially restore cell death after drug withdrawal. **(A)** In LAMA-R and **(B)** SUP-M2 LR Drugs were added at the indicated dose. Viability was assessed by trypan blue 5 days for LAMA-R and 3 days for SUP-M2-LR cells upon drug withdrawal, (**C**) ROS quantification was performed by confocal microscopy after 3 days or 1 day upon drug withdrawal for LAMA-R and SUP-M2 cells respectively.
